# Nanostructured MoS_2_ and WS_2_ Photoresponses under Gas Stimuli

**DOI:** 10.3390/nano12203585

**Published:** 2022-10-13

**Authors:** Mohamed A. Basyooni, Shrouk E. Zaki, Nada Alfryyan, Mohammed Tihtih, Yasin Ramazan Eker, Gamal F. Attia, Mücahit Yılmaz, Şule Ateş, Mohamed Shaban

**Affiliations:** 1 Department of Nanotechnology and Advanced Materials, Graduate School of Applied and Natural Science, Selçuk University, Konya 42030, Turkey; 2 Department of Nanoscience and Nanoengineering, Institute of Science and Technology, University of Necmettin Erbakan, Konya 42060, Turkey; 3 Theoretical Physics Department, National Research Center, Cairo 12622, Egypt; 4 Department of Physics, College of Sciences, Princess Nourah bint Abdulrahman University, P.O. Box 84428, Riyadh 11671, Saudi Arabia; 5 Institute of Ceramics and Polymer Engineering, University of Miskolc, H-3515 Miskolc, Hungary; 6 Department of Metallurgy and Material Engineering, Faculty of Engineering and Architecture, Necmettin Erbakan University, Konya 42060, Turkey; 7 Department of Solar and Space Research, National Research Institute of Astronomy and Geophysics (NRIAG), Cairo 11421, Egypt; 8 Department of Physics, Faculty of Science, Selçuk University, Konya 42075, Turkey; 9 Department of Physics, Faculty of Science, Islamic University of Madinah, P.O. Box 170, AlMadinah Almonawara 42351, Saudi Arabia; 10 Nanophotonics and Applications Laboratory, Physics Department, Faculty of Science, Beni-Suef University, Beni-Suef 62514, Egypt

**Keywords:** two-dimensional material, MoS_2_, WS_2_, thin film, optoelectronics

## Abstract

This study was on the optoelectronic properties of multilayered two-dimensional MoS_2_ and WS_2_ materials on a silicon substrate using sputtering physical vapor deposition (PVD) and chemical vapor deposition (CVD) techniques. For the first time, we report ultraviolet (UV) photoresponses under air, CO_2_, and O_2_ environments at different flow rates. The electrical Hall effect measurement showed the existence of MoS_2_ (n-type)/Si (p-type) and WS_2_ (P-type)/Si (p-type) heterojunctions with a higher sheet carrier concentration of 5.50 × 10^5^ cm^−2^ for WS_2_ thin film. The IV electrical results revealed that WS_2_ is more reactive than MoS_2_ film under different gas stimuli. WS_2_ film showed high stability under different bias voltages, even at zero bias voltage, due to the noticeably good carrier mobility of 29.8 × 10^2^ cm^2^/V. WS_2_ film indicated a fast rise/decay time of 0.23/0.21 s under air while a faster response of 0.190/0.10 s under a CO_2_ environment was observed. Additionally, the external quantum efficiency of WS_2_ revealed a remarkable enhancement in the CO_2_ environment of 1.62 × 10^8^ compared to MoS_2_ film with 6.74 × 10^6^. According to our findings, the presence of CO_2_ on the surface of WS_2_ improves such optoelectronic properties as photocurrent gain, photoresponsivity, external quantum efficiency, and detectivity. These results indicate potential applications of WS_2_ as a photodetector under gas stimuli for future optoelectronic applications.

## 1. Introduction

Photodetectors have a wide range of applications in the fields of biomedical sensing, environmental monitoring, optical communications, and space exploration. It is well known that photodetector responsivity is mainly dependent on the device material, structure, and operating conditions in terms of bias voltage, temperature, and wavelength of the incident radiation. It thus becomes crucial to have a thorough understanding of the effects of these parameters for designing and fabricating an optimal photodetector [1]. Nowadays, space has become a new and rich field, as well as a preferential development sector, which is rapidly contributing to countries’ financial welfare and progress. Space technologies play a key role in accelerating countries’ development processes and increasing societies’ quality of life and security. Aerospace and space applications require the development of sensors that are operated in several different environments. Future aeronautical systems will need to be more capable, perform better, and be safer, all of which will require less maintenance. Space sensors and detector application areas include optical, photodetector, leak detection, high temperature, emissions monitoring, fire detection, environmental monitoring, and radiation detection. Each of these detectors is the subject of effort throughout NASA to improve safety and decrease the cost of space travel, significantly reduce the number of emissions produced by aeronautic engines, and improve the safety of commercial airline travel. Testing the performance of the photodetectors under different gas environments is essential to understand their performances under various gas stimuli. For instance, CO_2_ is considered one of the primary gases in the space environment and the greenhouse gases in the Earth’s atmosphere [2,3,4]. The Martian atmosphere is primarily composed of 96% CO_2_ with a balance of nitrogen, argon, and trace species [5]. There is a highly significant need for testing the performance of optoelectronic devices under different gas environments, such as CO_2_, for space and commercial applications.

The exotic physical characteristics of 2D transition metal dichalcogenides (TMDC), such as their non-zero bandgap and layer-dependent second-order optical nonlinearity, are garnering a lot of interest. The chemical formula MX2 refers to a class of inorganic 2D layered materials where M is a transition metal (typically M = Mo, W, Ti, V, Ta, Hf, and Pt) and X is a group of VI chalcogen atom (typically X = S, Se, and Te). The advantages of both materials are that they can be combined by forming heterojunctions with silicon using 2D layered materials. By being completely compatible with conventional integrated circuit fabrication techniques, it streamlines the manufacturing process; because they have a bandgap of 2 eV in monolayer form. MoS_2_ and WS_2_ are the most studied materials in the TMDC family [6]. WS_2_ is more desirable for optical, electrical, and optoelectronic applications due to its novel properties, including high thermal stability and a wide range of operating temperatures [7], layer-dependent tunable bandgap (1.4–2.1 eV) [8], broad UV-visible absorption spectrum [9], and tunable photoluminescence (PL) effect [10,11,12].

Graphene, WS_2_, MoS_2_, and other materials can be produced into 2D sheets using “top-down” techniques like exfoliation methods. Among these, micromechanical exfoliation [13], sonication-assisted liquid exfoliation [14,15,16], shear exfoliation [17,18], and chemical exfoliation [19,20] have been studied and suggested in the literature. However, they have several disadvantages, such as low quality and small-scale production, many flaws, and a short-range during micromechanical exfoliation [21]. Additionally, transferring the exfoliated layer to a new substrate is required, which makes scaling up and large-scale production more difficult [22]. For this reason, chemical vapor deposition (CVD) with a bottom-up process is preferred during the synthesis of MoS_2_ [23,24]. MoO_3_ substrate is maintained in the downstream gas flow during growth [25,26,27]. Thin-film properties like homogeneity and grain size are strongly dependent on MoO_3_ substrate properties. Nowadays, the creation of wafer-scale and homogeneous 2D materials has attracted great attention due to the advancement of the following generation of optoelectronic applications and quantum computers. Atomic layer deposition (ALD) [22,28], pulsed laser deposition (PLD) [29,30], thermal evaporation [31,32,33], and magnetron sputtering systems [34,35,36] are commonly used for the preparation of MoO_3_ substrates. However, magnetron sputtering is the most preferred, since its low cost and ease of control are suitable for large-scale commercial manufacturing. MoO_3_ is sulfurized within a two or three-zone quartz tube under an Ar inert atmosphere via sublimation of sulfur powders. Similar processes are used with WO_3_ to grow WS_2_ TMDC.

WS_2_ and MoS_2_ are two TMDC thin films that have tunable optical bandgaps, making them promising for photodetection applications. A suitable structure may be sensitive to an incident light involving a photocurrent whose intensity is compared to the current under darkness (dark current) [37]. Photoconduction and photogate are two major effects that contribute to photocurrent [38]. The process of photoconduction happens when free charge carriers are absorbed by light with photon energy greater than the MoS_2_ (or WS_2_) bandgap. The highest photo responsiveness occurs when all photons are absorbed and each input photon produces one electron and one hole. This indicates that the primary photoresponse mechanism in MoS_2_ (or WS_2_) phototransistors is a photogate, in which light absorption introduced a change in the trapped charges density. When photogate occurs in the MoS_2_ (or WS_2_) thin film, the threshold voltage V_TH_, the gate voltage separating the high-current (ON) and low-current (OFF) regimes, shifts as a result of trapped charges causing a change in the effective gate voltage, which introduces a great increase in current [39].

Gas adsorption can affect the Fermi-level energy of MoS_2_ [40]; therefore, several investigations on 2D TMDC-based photodetectors and gas sensors have been conducted [41]. The effect of O_2_, N_2_, and Ar on the optoelectronic rGO performance was studied [42]. As with n-type rGO, oxidizing gases such as O_2_, CO_2_, and N_2_ pull electron clouds from the n-type semiconductor sheets, resulting in a drop in charge carriers and an increase in resistance [42,43]. Different compounds have also been tested, such as the effect of the atmosphere on the device performance of perovskite solar cells during operation. Indeed, Guo et al. [44] investigated the degradation mechanisms of perovskite solar cells operated under a vacuum and a nitrogen atmosphere.

According to our investigations, there is no complete work related to the study of the effect of CO_2_ gas adsorptions on MoS_2_ and WS_2_ by examining and comparing photodetection parameters. To fabricate MoS_2_ and WS_2_-based optoelectronic photodetector sensors, PVD layers of molybdenum and tungsten oxide were deposited on silicon substrates, then sulfurization occurred in a CVD chamber. The effect of different gas adsorptions, such as air, O_2_, and CO_2_, on MoS_2_ and WS_2_ thin films, were investigated in this work. In addition, their optoelectronic performance under UV illumination is discussed in detail.

## 2. Materials and Fabrication Methods

### 2.1. Fabrication of Thin Films

The fabrication process of MoS_2_ and WS_2_ thin films is similar to the previously reported studies [45,46]. The advantage of this process is to increase film homogeneity and scalability. This method also demands that there are no small discounted triangular-like shapes of MoS_2_ or WS_2_, as known for the CVD deposition process of 2D materials. MoS_2_ and WS_2_ thin films were prepared on a p-type silicon substrate first by using a PVD system, which included an RF magnetron sputtering system followed by a CVD process. Substrates of Si were cleaned in a series of steps, firstly a 5 min dip in an NH_4_OH-H_2_O_2_ solution watered down with pure water at 75 °C. Then, they were put in a 5% HF solution for 5 s, after which they were washed in pure water and dried with N_2_. The Si substrates were put into the RF magnetron sputtering system right away. To activate the Si surface, a 100 W Ar-plasma source was opened for 10 min at room temperature. Targets made of tungsten and molybdenum were used as the primary sources, with Ar plasma serving as the carrier gas and O_2_ as the reactive gas. The substrate temperature was kept under control at 400 °C for more than 30 min, increasing by 100 °C/30 min. We maintained constant O_2_ and Ar flow rates. The films were deposited at 5E-3 Torr and 137 W using 30 s sputtering times. Before being moved to the two-zone CVD quartz chamber for the sulfurization process, the system was naturally cooled to room temperature. In the center of the CVD furnace, the temperature of the Mo-O and W-O thin films as they were being deposited was elevated to 650–750 °C. A 100 sccm Ar source was used with a ceramic boot that contained 0.5 g sulfur powder. An external heating belt was used to evaporate the sulfur for 25 min at a distance of 50 cm from the substrate. The system then cooled until it reached RT while receiving an Ar flow rate of 100 sccm.

### 2.2. Characterization Techniques

**Scheme 1:**
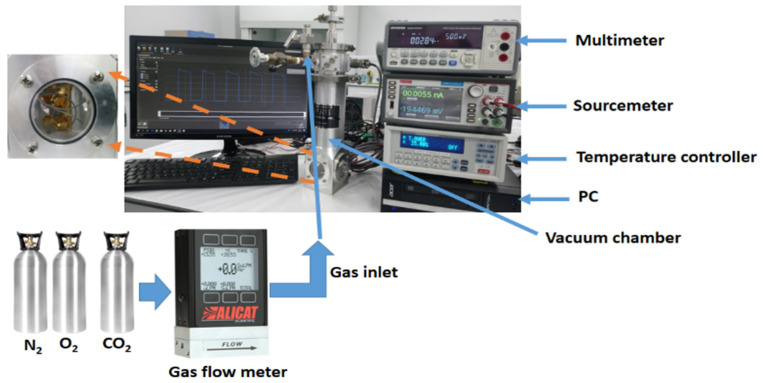
Electrical and optoelectrical systems under different gas environments and different temperature conditions.

Field-emission scanning electron microscopy (FE-SEM; Zeiss Gemini 500, Cambridge, UK) was used to record the surface morphology. The topography and line profile spectrum were examined using atomic force microscopy (AFM, Park, Santa Clara, CA, USA) Park XE7 system via noncontact mode through a 1 × 1 µm scanning area and a tip scan speed of 1 Hz. XEI 4.3.4 2016 data processing and analysis software (Park, Santa Clara, CA, USA) were used to measure the roughness values. In addition, confocal Raman microscopy was utilized to introduce thin film optical images. X-ray photoelectron spectroscopy (XPS) measurements were investigated based on the Thermo Scientific K-alpha XPS system (Thermo Scientific™, Waltham, MA, USA) using an Al K_α_ source and a spot size of 400 µm. Photoluminescence (PL) and Raman vibrational modes were performed using Renishaw inVia confocal Raman microscope (Renishaw, New Mills, UK) with a laser beam of a wavelength value of 532 nm. SWIN Hall8800 Hall Effect measurement system is employed to measure the carrier’s concentration and mobility. The electrical I-V and optoelectronics measurements were carried out using Tektronix Keithley 2400 Sourcemeter (20 mV and 10 nA sensitive, Tektronix, Beaverton, OR, USA) through a four-probe system and KickStart Keithley software (Tektronix, Beaverton, OR, USA) for data acquisitions. A 365 nm ultraviolet (UV, Konya, Turkey) light lamp was used for illumination. The same calculations and measurements can be reported under different gas (such as CO_2_) and temperature environments by using a stain steel vacuum chamber with a quartz window, as shown in Scheme 1. The system has a quartz window that can be used to transmit light into the sample easily under temperature or gas environments. The temperature can be controlled with a Lake Shore Model 335 Cryogenic Temperature Controller (Woburn, MA, USA). These measurements were repeated under high-purity gases of O_2_, CO_2,_ and N_2_ to study the stimuli effects. Here, the role of N_2_ was just to clean/stabilize the environment around the photodetector sample. Since we were using different gases (CO_2_ and O_2_), cleaning the environment around the sample with a vacuum and inert gas (N_2_) was necessary. The gases are controlled with the Alicat Scientific Mass flow meter MC model. All the measurements were recorded and captured from the computer, and after that, the analyses were carried out.

## 3. Results and Discussion

### 3.1. Raman and PL Spectra

**Figure 1:**
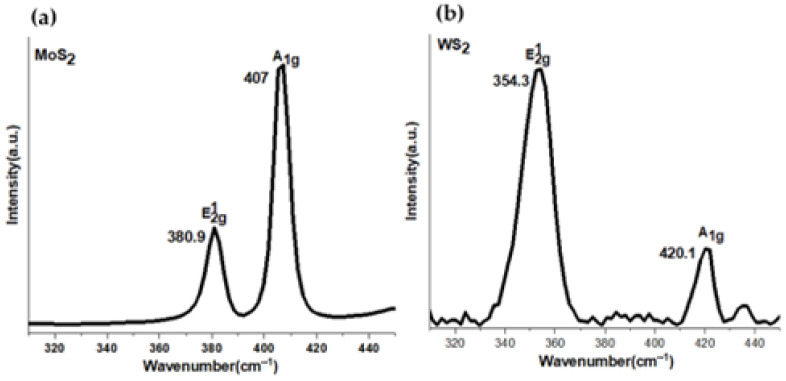
(**a**,**b**) Raman spectra of MoS_2_ and WS_2_ on Si substrates, respectively.

The Raman spectra of 2D materials show characteristics of two main peaks with $E_{2g}^{1}$ and $A_{1g}\;$ modes. According to the literature, Mo–S atoms vibrate in the plane at $E_{2g}^{1}$ = 382.01 cm^−1^ while S atoms oscillate perpendicular to the plane at $A_{1g}$ = 407 cm^−1^, as shown in Figure 1a [23,24,47]. Concerning WS_2_, $E_{2g}^{1}$ and $A_{1g}$ modes, the peaks are located at 354.08 and 420.09 cm^−1^, respectively. The differences between $E_{2g}^{1}$ and $A_{1g}$ for MoS_2_ and WS_2_ are 23.76 and 66.01 cm^−1^, respectively. These results show the existence of multilayered MoS_2_ and WS_2_ structures of a few nanometers in size, as has been reported before [24,45,48].

**Figure 2:**
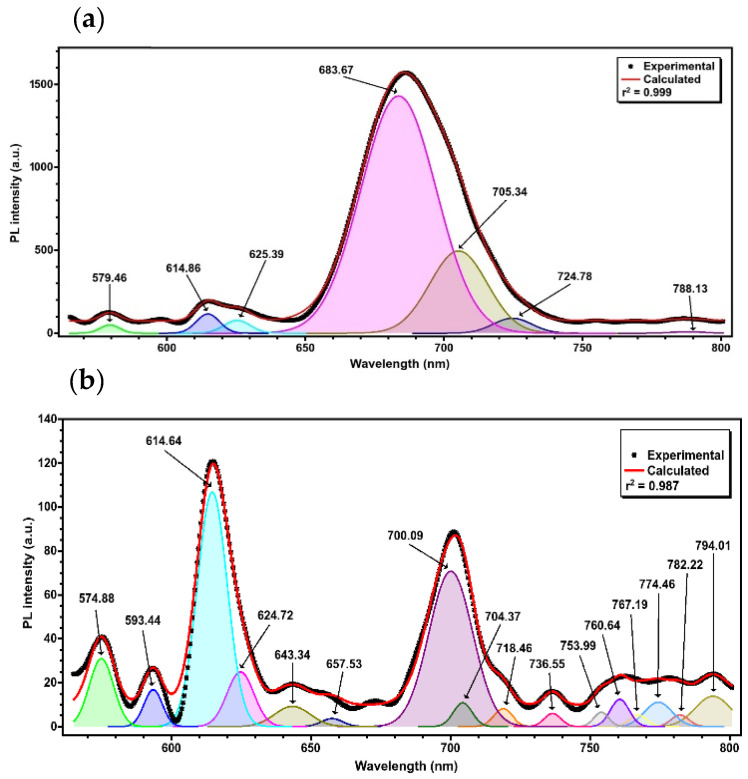
(**a**,**b**) PL spectra of MoS_2_ and WS_2_ on Si substrates, respectively.

From the measurements of photoluminescence (PL), it can be seen that the thin films introduced a direct and indirect electronic band transition. MoS_2_′s optical properties are greatly influenced by the number of layers. The energies of direct excitonic transitions have a substantial PL effect in monolayer MoS_2_, but this effect is reduced due to the layer’s number increasing and completely disappearing in bulk MoS_2_. The PL spectra of MoS_2_ are observed at 610 and 624 nm (Figure 2a). These peaks are related to direct excitonic transitions at the Brillouin zone of the K point and represent the A and B excitation of MoS_2_ [49]. Furthermore, photogenerated electron-hole pair recombination is attributed to the prominent peak at 682 nm, while the valence band separated as the MoS_2_^’^s high spin-orbit coupling is attributed to the lesser peak at 624 nm [50]. Nevertheless, the trion exhibits localized quasiparticles with negative (two electrons with one hole) and positive (two holes with one electron) charges as PL peaks at 682 nm. According to the report, the monolayer introduced a strong peak at 615 nm (2.02 eV) that was associated with the A exciton resonance. It is believed that at the K point, the direct exciton transition between the maximum of the valence band and the minimum of the conduction band (CBM) is what causes the PL in the monolayer [51]. However, the bilayer and trilayer PL spectra show wider and lower energy emissions than those of the indirect exciton, in which the VBM is still at the K point but the CBM is located between the K point and the peaks [52]. For WS_2_, two peaks at 612 and 700 nm are observed (Figure 2b).

### 3.2. Surface Morphology and Topography

**Figure 3:**
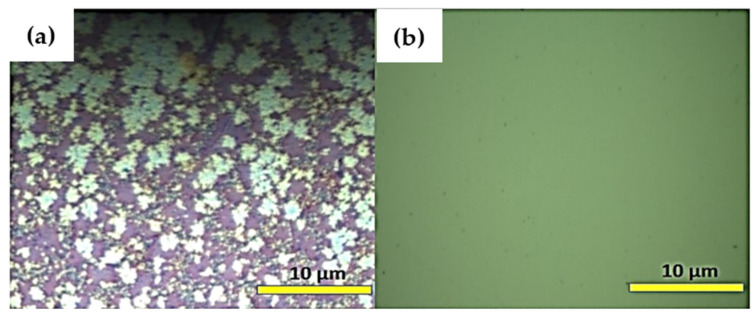
(**a**) MoS_2_ and (**b**) WS_2_ thin-film optical microscopy images.

Large-scale MoS_2_ thin films have been previously worked on by combining CVD and sputtering techniques [53]. Optical microscopy was used to capture the MoS_2_ and WS_2_ surfaces, as seen in Figure 3a,b, respectively. At a macroscopic scale, the texture of WS_2_ is more homogeneous than that of MoS_2_. The structural domains of WS_2_ are smaller than those of MoS_2_ (see Figure 3b).

**Figure 4:**
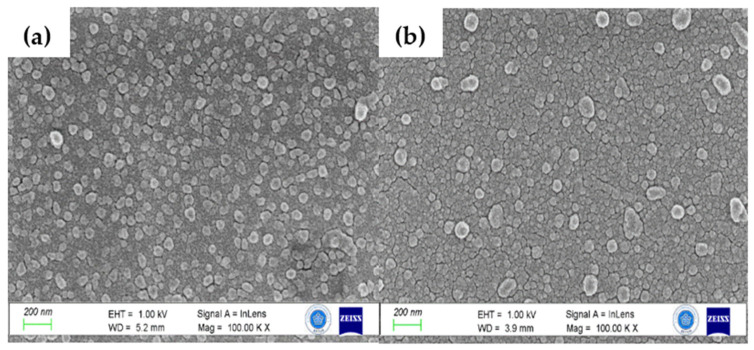
(**a**,**b**) FE-SEM morphology of MoS_2_ and WS_2_ thin films.

The high-resolution FE-SEM images show quite a homogeneous distribution of a few nanometers that contain nanocrystals for both materials (Figure 4). However, the particles of MoS_2_ are relatively smaller than those of WS_2_. In both materials, particles are well interconnected to each other. Nevertheless, the WS_2_ surface contains more cracks than MoS_2_, which is important for surface gas adsorption and light-trapping applications [54].

**Figure 5:**
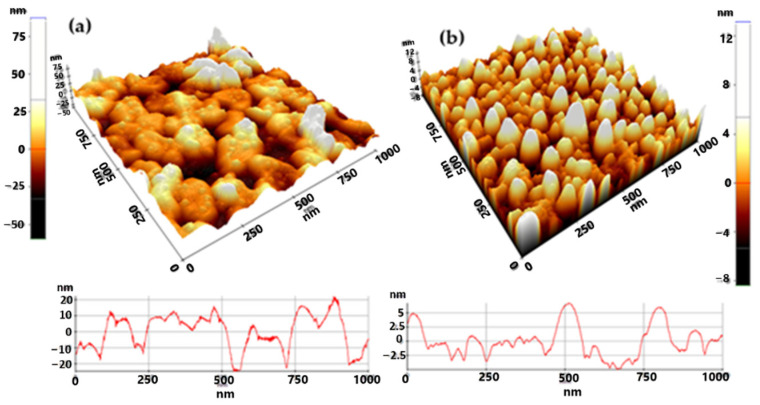
AFM topography and the horizontal line profile: (**a**,**b**) 3D topography images and corresponding line profile of MoS_2_ and WS_2_ samples, respectively.

The topography of the prepared samples was measured by AFM using a noncontact mode of a 1 × 1 μm area. Figure 5a,b show the 3D AFM topography and the horizontal line profile of MoS_2_ and WS_2_ samples, respectively. The roughness values of MoS_2_ and WS_2_ are 10.689 and 2.761 nm, respectively, as depicted in Table 1. The optical microscopy analysis also shows that MoS_2_ has higher surface roughness than WS_2_ film.

### 3.3. XPS and Oxidation States

**Figure 6:**
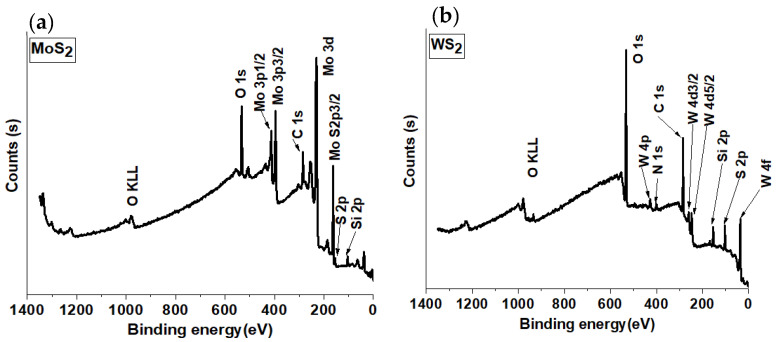
(**a**,**b**) General XPS survey spectrum distributions of both MoS_2_ and WS_2_ thin films.

The WS_2_ and MoS_2_ chemical states were examined through XPS survey analyses. The binding energies of S 2p, Mo 3d, C 1s, and O 1s are introduced by the characteristic peaks at 161 eV, 230 eV, 285 eV, and 532 eV, respectively [55], as shown in Figure 6a. The XPS survey analyses of WS_2_ showed distinct signals of C, O, S, and W elements, as seen in Figure 6b. The W 4d_3/2_ spin-orbital splitting photoelectrons in the WS_2_ nanostructures are reconsidering the W peak at 283.0 eV [56]. In addition, the W 4d_3/2_ peak binding energy is higher than the W atom, which confirms the presence of W with a valence of +4 [57].

### 3.4. Sheet Resistance and Hall Coefficient

Electrical characterization of the prepared MoS_2_ and WS_2_ thin films was carried out using a Hall-effect measurement system (Table 1). Table 2 displays the R_s_ (sheet resistance), R_ho_ (resistivity), V_H_ (Hall voltage), R_H_ (Hall coefficient), N_s_/P_s_ (sheet carrier concentration), N/P (carrier concentration), and Mob (mobility). When V_H_ has a positive (negative) value, the majority of the sample’s carriers are of the p (n) type. All measurements were performed with a 7210 G magnetic field and a temperature of 300 K. We observed an enhancement in the electrical mobility properties of the WS_2_ compared to the MoS_2_ thin film. The MoS_2_ film shows an n-type and WS_2_ is p-type semiconductor behavior [58]. Based on these investigations of Hall-effect measurements, it was observed that our devices are MoS_2_ (n-type)/Si (p-type) and WS_2_ (P-type)/Si (p-type) heterojunctions. The highest carrier concentration, as well as carrier mobility for p-p heterostructure, was higher than that of the p-n device, suggesting better performance for the WS_2_/Si device.

### 3.5. Electric and Optoelectronic Characteristics

**Figure 7:**
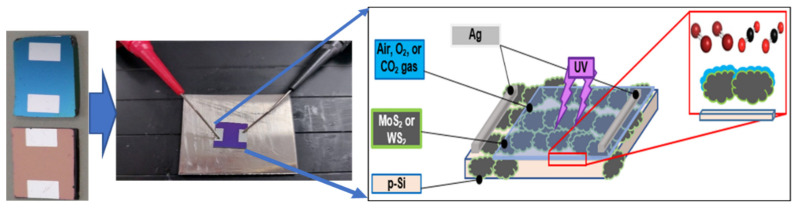
The MoS_2_ and WS_2_ photodetector under the illumination of UV light and the device’s actual image as seen through the probe station. The measurements were carried out in the air, O_2_, and CO_2_ environments.

The current-voltage (I-V) electrical characteristics of MoS_2_ and WS_2_ were examined under air conditions in absence of light and 365 nm ultraviolet illuminations. The device size was 1 × 1 cm^2^, and we used silver paste for making the contacts with a width of 3 mm and a length of 1 cm. The vertical electron transfer (electrical and optoelectronic) multilayered MoS_2_/Si and WS_2_/Si heterostructures were investigated and active edge sites with high density [59] are shown in Figure 7. As mentioned above, a high resistance layer like SiO_2_ [60], which increased the ideal structure vertical conductivity, is not presented in the currently optimized photodetector.

**Figure 8:**
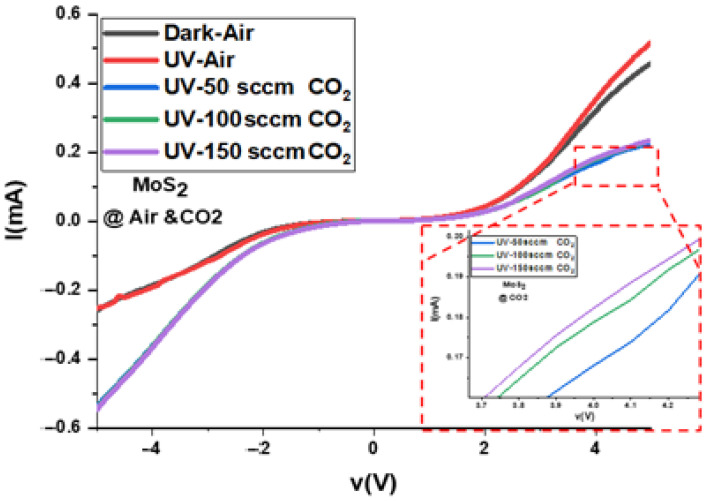
I-V characteristic of MoS_2_ in the absence of light and UV illumination conditions under air and CO_2_.

MoS_2_: The idea of measuring the optoelectronic performance of devices under different gas stimuli, such as CO_2_, has received much interest for many environmental and industrial applications. The behavior of optoelectronic photodetectors under a toxic gas environment such as CO_2_ may predict the general performance of devices. Figure 8 shows the I-V curve of MoS_2_ in dark and UV conditions of 365 nm under air and CO_2_ gas stimuli. Under dark and illumination conditions, nonlinear I-V curves were observed, showing the generation of an excellent double-Schottky contact between the silver (Ag) electrode and film surfaces, as predicted before [61]. The observed photocurrent is small compared to the dark current in the air. Under CO_2_ gas flow, the photocurrent is lower than the one under air in both positive and negative parts. This result shows the n-type behavior of MoS_2_ under CO_2_. CO_2_ gas can capture the electrons from the conduction band of the n-type MoS_2_ surface, which consequently can decrease the current. Since CO_2_ does not absorb light at 365 nm, the reduction in photocurrent is related to its oxidizing properties that act on the MoS_2_ surface. Similar behavior has been predicted before for different n-type semiconductor materials [42,43]. Moreover, by increasing the CO_2_ gas flow from 50 to 150 sccm, a further but less pronounced photocurrent decrease is observed, supporting the effect of oxidation.

**Figure 9:**
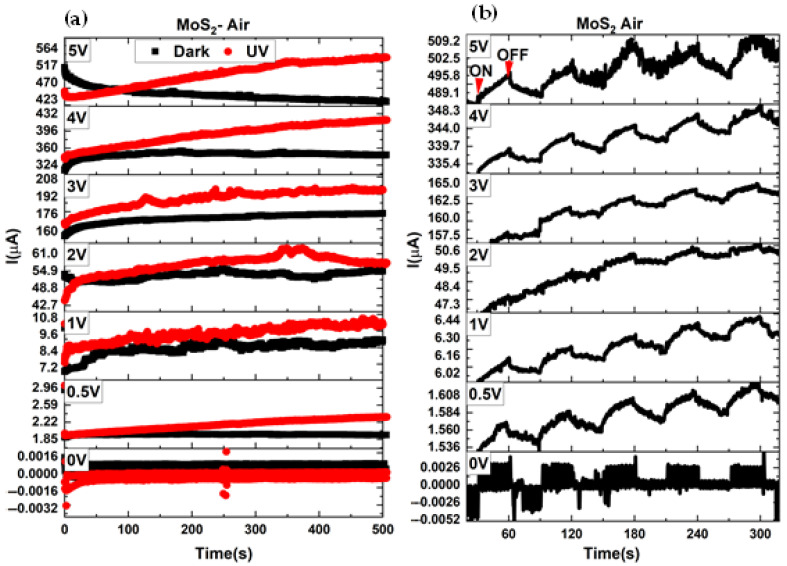
(**a**) Photocurrent behavior with time of MoS_2_ under dark and UV illumination conditions in air, and (**b**) the on–off time-resolved photoresponse of MoS_2_ thin film under bias voltages of 0–5 V.

To check the behavior of the MoS_2_ photodetector with time, we measured the change in the photocurrent in the air under dark and light illumination conditions, as shown in Figure 9a. The measurements are carried out at different bias voltages from 0 to 5 V. The MoS_2_ in air did not show a good response at zero bias, but the response started to appear from 0.5 V. With increased applied bias, the sensor performance becomes dominated and more stable and produces higher photocurrent. Figure 9b shows the on–off time-resolved photoresponse of MoS_2_ thin film under a different bias voltage of 0–5 V. The measurements were recorded every 30 s and started with dark mode followed by the UV-on illumination mode. The dynamic curve of the MoS_2_ at zero bias did not show a good signal, confirming that it is not a self-bias photodetector. However, with a small bias of 0.5 V, a clear signal gets released. We see that at higher applied biases, the device is working more efficiently, as expected.

**Figure 10:**
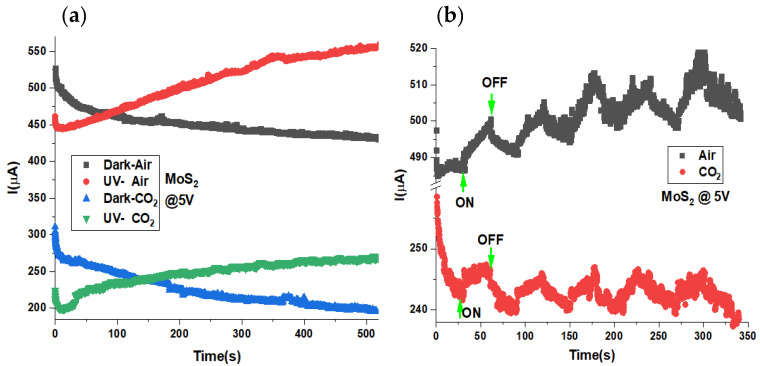
(**a**,**b**) Photocurrent behavior with time and on–off time-resolved photoresponse of MoS_2_ thin film under dark and UV illumination conditions in both air and 50 sccm CO_2_ environments at a bias voltage of 5 V.

To check the photocurrent time and the on–off behavior of the MoS_2_ photodetector in air and CO_2_, we chose a bias of 5 V. Figure 10a,b show the photocurrent behavior with time and on–off time-resolved photoresponse of MoS_2_ thin film under the absence of light and UV illumination conditions in both air and 50 sccm CO_2_ environments at a bias voltage of 5 V. The behavior of the photocurrent in both cases (air and CO_2_) is more or less the same. Therefore, in the case of CO_2_, we see that the dark current is decaying with time but the photocurrent is increasing, similar to the case of air. However, the decreasing and increasing rate of the current for the case of CO_2_ stimuli is less than in the case of air, confirming the better stability of the MoS_2_ photodetector in CO_2_ than in an air environment. The position of the CO_2_ curve is lower than that of the air, due to the capturing mechanism of the CO_2_ of electrons and subsequently increasing the resistance. On the other hand, for the on–off curve in Figure 10b, the MoS_2_ photodetector in CO_2_ long-term stability with time is better than in the case of air. In both cases, the response and recovery time are relatively long.

**Figure 11:**
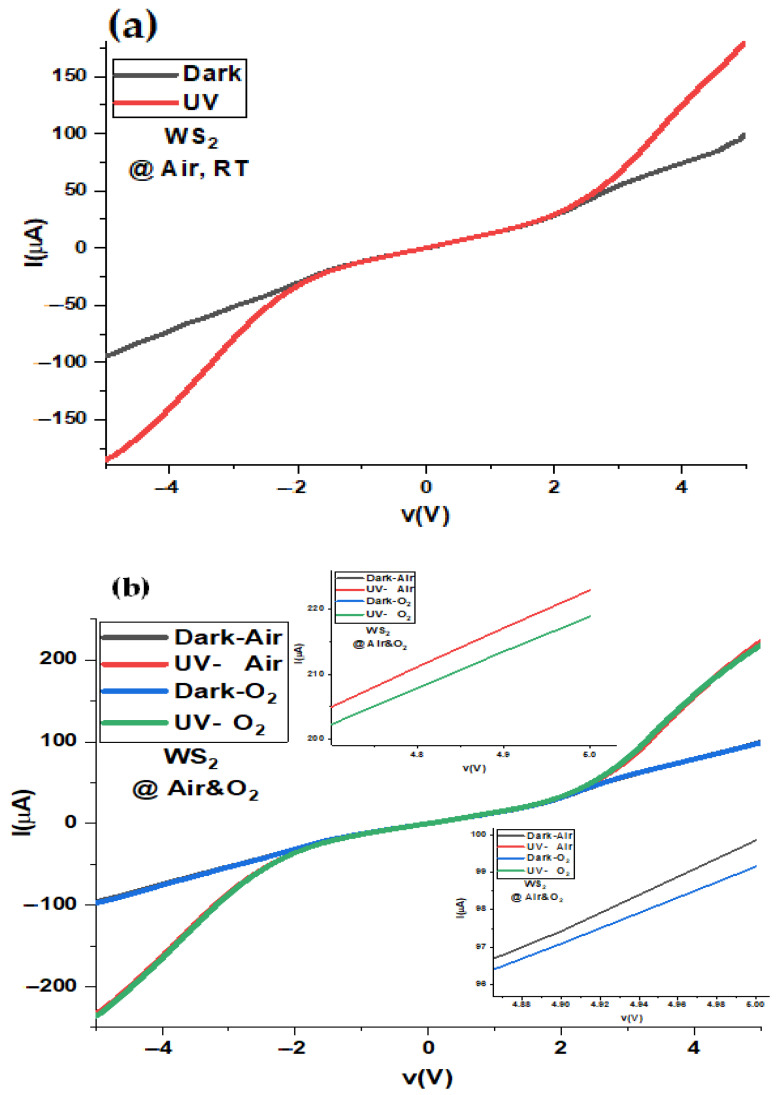

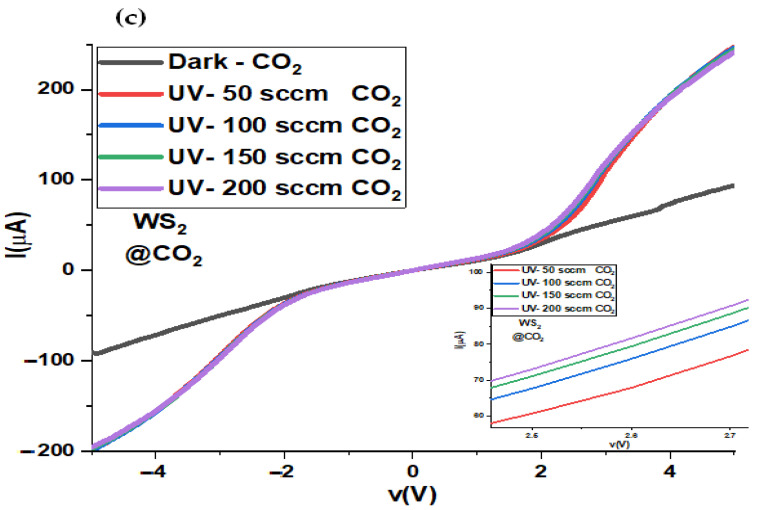
Characteristic I-V curve of WS_2_ under dark and UV illumination conditions under (**a**) air environment, (**b**) O_2_ environment and (**c**) CO_2_ environment.

On the other hand, for WS_2_, we observed a better response than MoS_2_ thin film. We measured the electrical IV properties of WS_2_ thin film under dark and UV illuminations in air, O_2_, and CO_2_ environments, as in Figure 11. The IV curve in air is illustrated in Figure 11a. We observed a better response under light illumination in both positive and negative parts than in the case of MoS_2_ thin film. However, for the IV of MoS_2_ above, only a small improvement in the positive current was shown, but there were no changes in the negative section. This indicated that WS_2_ is more interesting for optoelectronic applications, so we tested WS_2_ film under O_2_ and CO_2_ environments. In the case of 100 sccm O_2_ stimuli, its photocurrent is decreasing for both dark and UV cases, as represented in Figure 11b. This shows that the Fermi level’s position has a substantial effect on O_2_ and CO_2_ molecule adsorption and desorption at the surface. Charge transfer is expected to attract electrons from the p-type WS_2_ layer because CO_2_ is an electronegative molecule [62].

**Figure 12:**
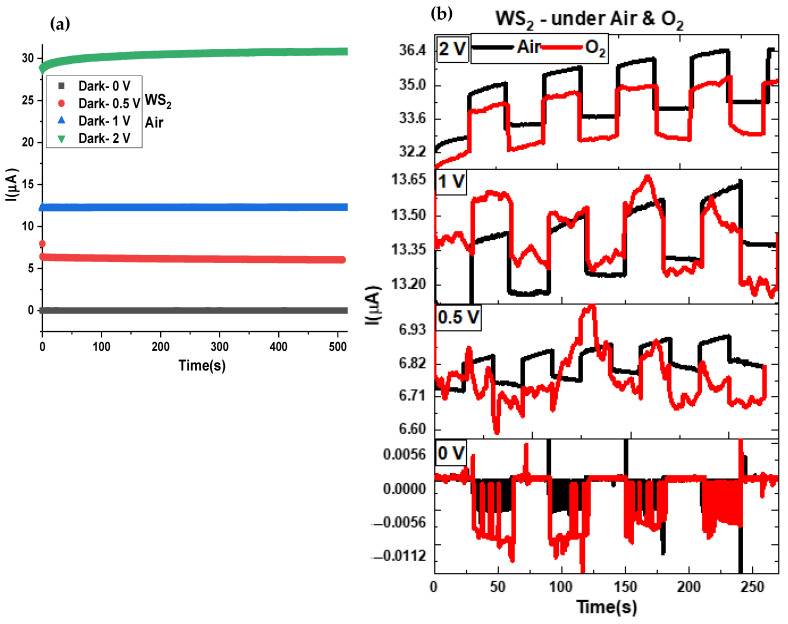
(**a**) Photocurrent behavior with the time of WS_2_ thin film under a dark condition in the air at a different bias voltage of 0, 0.5, 1, and 2 V. (**b**) The on–off dynamics of the current with a time of WS_2_ thin film in air and 50 sccm O_2_ environments under a different bias voltage of 0–2 V.

Normally, O_2_ and CO_2_ are oxidizing gases due to their high affinity for electrons and high electronegativity. However, in the case of CO_2_ molecules, the electronegativity is stronger than in the O_2_ case. We observed an increase in the photocurrent under the CO_2_ environment in Figure 11c. The photocurrent is increased with increasing the CO_2_ concentrations from 50 to 200 sccm. The p-type semiconductor behavior of WS2 under CO2 adsorption increases the total current as observed here in Figure 11. To check the WS_2_ film stability under air, we measured the photocurrent behavior as a function of time of WS_2_ thin film under the dark condition in the air at a different bias voltage of 0, 0.5, 1, and 2 V, as demonstrated in Figure 12a. In the case of WS_2_, we found that the film became more stable at low bias voltage, in contrast to the case of MoS_2_, so we did not test the WS_2_ films at higher bias voltage. By increasing the bias voltage, the current was increased. However, by applying 2 V, the current reached higher values. Consequently, the on–off behavior of WS_2_ thin film was tested under the same applied biases of 0, 0.5, 1, and 2 V and is depicted in Figure 12b. At 0 V bias, for the dark current in the air, almost no current is observed with some signal noises. But under O_2_ stimuli, we observed a negative current behavior as in Figure 12b, indicating that WS_2_ can work as a negative photoresponse optical detector under low biases [40]. However, at 0.5, 1, and 2 V bias, the current becomes positive for under air and O_2_ environments. At 0.5 and 1 V, there were almost no changes in the current under air and O_2_ gas environments. The best current response was observed at 2 V for both air and O_2_ environments with the same behavior, as expected (Figure 12b).

**Figure 13:**
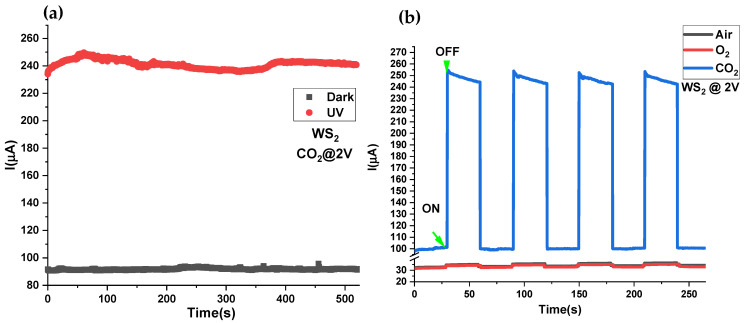
(**a**) Photocurrent behavior with time of WS_2_ thin film under dark and UV conditions in CO_2_ at a bias voltage of 2 V. (**b**) The on–off dynamics of the photoresponse with a time of WS_2_ thin film in air, 50 sccm O_2_, and 50 sccm CO_2_ environments under a bias voltage of 2 V.

By these means, we measured the photocurrent of WS_2_ at 2 V under air and CO_2_. Figure 13a show the photocurrent behavior with a time of WS_2_ thin film under dark and UV conditions in CO_2_ at a bias voltage of 2 V. We see that the general behavior is positive photoresponse. The demonstrated results show that WS_2_ film presented long-term stability in CO_2_ and also in the air with time. Figure 13b shows the on–off photoresponse dynamics of WS_2_ thin film in air, 50 sccm O_2_, and 50 sccm CO_2_ environments under a bias voltage of 2 V. The photocurrent of WS_2_ thin film is largely enhanced by introducing CO_2_ gas under UV illumination. In contrast, for the air and O_2_ environment, we do not see a large enhancement in the photocurrent. The on–off curve shows high stability during many pulses. Under the same conditions, the WS_2_ introduced a higher sensitivity to environmental gases including CO_2_ and O_2_ than MoS_2_ thin film. In addition, we found that the high photocurrent is shown under the CO_2_ stimuli for WS_2_ under various gas conditions.

### 3.6. Transient Response

**Figure 14:**
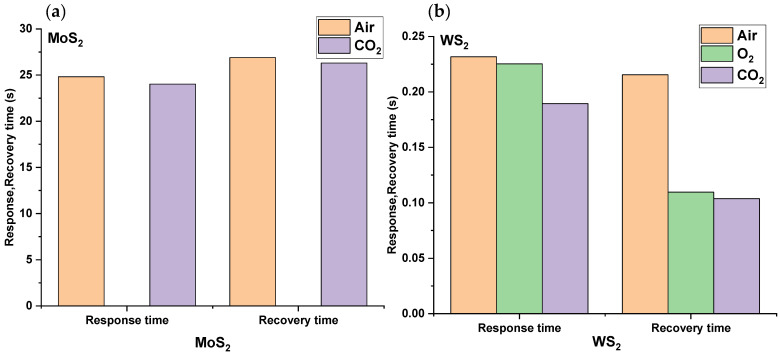
Shows the response and therecovery times for (**a**) MoS_2_ and (**b**) WS_2_ thin films under gas stimuli.

The MoS_2_ and WS_2_ thin-film response and recovery time under UV illumination of 365 nm are c depicted in Figure 14. The MoS_2_ and WS_2_ on–off behavior gives us a sense of the rise and fall with time under dark and illumination conditions. The response/rise time was calculated when the source of light opened, and when the light was turned off, the recovery/decay time was measured. The MoS_2_ thin film has a longer recovery time and faster response time in an air environment, as in Figure 14a. However, under CO_2_ stimuli, a longer response/recovery time is noted. The longer response time may refer to the CO_2_ gas adsorption on MoS_2_, which is faster than the desorption kinetics. The observed response/recovery time of MoS_2_ is in seconds, which limits the application of the MoS_2_ photodetector. However, for the WS_2_ thin film, a faster response is observed, as in Figure 14b. Generally, the WS_2_ thin film introduced a shorter response time than the recovery time. In addition, shorter response and recovery times are observed for the CO_2_ case compared with air and O_2_ stimuli. The CO_2_ adsorption/desorption kinetics on WS_2_ are faster than in the air and O_2_ gas cases. The adsorption energy and diffusion coefficient of O_2_ were the lowest [63].

### 3.7. Photocurrent Gain (P_g_), Photoresponsivity ($R_{\lambda }$), External Quantum Efficiency (EQE) and Detectivity ($D^{*}$)

Some other parameters may contribute to the general performance of the photodetector sensor, such as photocurrent gain (P_g_), photoresponsivity ($R_{\lambda }$), external quantum efficiency (EQE), and detectivity ($D^{*}$). $I_{\mathrm{ph}}=I_{\mathrm{Light}}-I_{\mathrm{Dark}}$ gives the induced photocurrent $I_{\mathrm{ph}}$, where $I_{\mathrm{ph}}$ increases as the applied voltage and light power increase [64]. Here, the light intensity is maintained at a consistent level and excitation wavelength of 365 nm. Photocurrent gain (P_g_), responsivity ($R_{\lambda }$), and external quantum efficiency can be calculated as reported before [23,24,65,66]. The detectable signal is another significant figure of merit for a photodetector referred by the specific detectivity [67]. The parameters of $I_{\mathrm{ph}}$, P_g_, $R_{\lambda }$, EQE, and $D^{*}$ for MoS_2_ and WS_2_ thin films are reported in Table 3. It seems that the general performance of MoS_2_ is weaker than WS_2_ thin film. The $I_{\mathrm{ph}}$ of MoS_2_ is decreased by introducing CO_2_ as explained in Figure 8. Consequently, all the other parameters will get affected in a similar way. The EQE of MoS_2_ thin film under air is higher than that in CO_2_.

**Figure 15:**
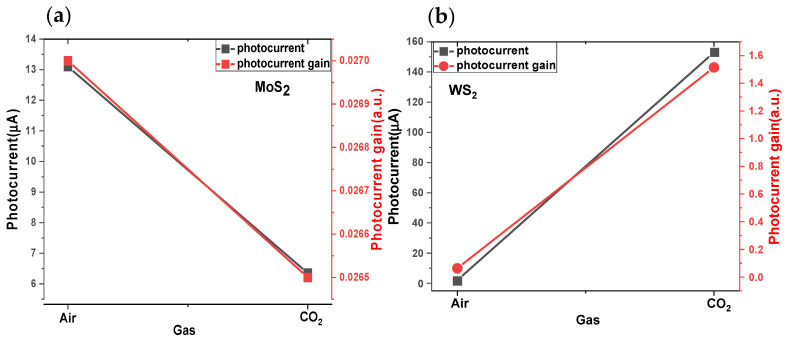
(**a**,**b**) Photocurrent and photocurrent gain of MoS_2_ and WS_2_ thin film under gas stimuli.

The photocurrent and photocurrent gain of MoS_2_ and WS_2_ under air and CO_2_ are represented in Figure 15. For WS_2_ film, a clear enhancement in both photocurrent and photocurrent gain is observed under CO_2_ stimuli. In the case of WS_2_ thin film, we introduced a p-type semiconductor with a large carrier concentration value of 5,500,000,000,000 cm^−2^; consequently, a high photocurrent is observed in the air. We observed a large enhancement in the EQE in the air of 2,200,000 compared with CO_2_ of 162,000,000, due to the fact that CO_2_ is an oxidizing agent interacting with p-type semiconductor materials, which can improve the electron concentrations and increase the conductivity of the film, as in Figure 15b [68]. Under O_2_ stimuli, we observed a lower responsivity of 0.915 µA/mW. On the other hand, for the MoS_2_ thin film, we have an n-type semiconductor that is interacting with oxidizing CO_2_ gas, which will increase the film resistance and consequently decrease the photocurrent, as in Figure 15a.

## 4. Conclusions

A 2D transition metal dichalcogenide heterojunctions of MoS_2_ and WS_2_ on silicon substrates for optoelectronic applications have been introduced. Using commercial chemical and physical vapor deposition techniques, we combined them for large-scale photodetector applications and beyond. For the first time, we exposed CO_2_ and O_2_ gases through a designed chamber to test their effects on the UV photodetector applications. The semiconducting behavior of MoS_2_ and WS_2_ thin films are n- and p-type with sheet carrier concentrations of 3,500,000,000,000 and 5,500,000,000,000 cm^−2^. The WS_2_ thin film showed higher carrier mobility of 2980 cm^2^/V compared to 946 cm^2^/V of MoS_2_ film. WS_2_ showed a fast response under UV illumination than MoS_2_ under air, CO_2_, and O_2_ environments. The calculated detectivity of WS_2_ showed higher values compared to the air and O_2_ adsorbed gases. We also observed that the EQE of WS_2_ under CO_2_ is 162,000,000 compared with 6,740,000 for the case of MoS_2_ thin film. Our findings provide high motivation for using MoS_2_ and WS_2_ thin films for space and industrial applications filled with environmental gases.zzzz

## Figures and Tables

**Table 1:** AFM roughness parameters of MoS_2_ and WS_2_ samples.

Sample	Region	Min (nm)	Max (nm)	Mid (nm)	R_q_ (nm)	R_a_ (nm)	R_z_ (nm)	R_sk_	R_ku_
MoS_2_	Line	−24.584	22.080	−1.252	46.664	10.689	9.200	45.329	0.439
WS_2_	Line	−4.964	6.682	0.859	11.646	2.761	2.099	11.374	−0.645

**Table 2:** Room-temperature surface resistance and Hall-effect measurements for MoS_2_ and WS_2_ on a silicon substrate.

Sample	R_s_ (Ω/sq)	R_ho_ (/Ω-cm)	V_H_ (V)	R_H_ (m^3^/C)	Type	N_s_/P_s_ (/cm^2^)	N/P (/cm^3^)	Mobility (cm^2^/V)
MoS_2_	1890	0.00284	−0.0129	−0.00000268	N	−3,500,000,000,000	−2,330,000,000,000,000,000	946
WS_2_	38,200	0.0573	0.00819	0.00000171	P	5,500,000,000,000	3,670,000,000,000,000,000	2980

**Table 3:** The photocurrent ($I_{\mathrm{ph}}$), photocurrent gain (P_g_), photoresponsivity ($R_{\lambda }$), external quantum efficiency (EQE), and detectivity ($D^{*}$) of MoS_2_ and WS_2_ samples.

Sample	Gas	Photocurrent (A)	Photocurrent Gain (a.u.)	Responsivity (µA/mW)	EQE	$D^{*}$
MoS_2_	Air	0.0000131	0.0270	6.5745	13,900,000	372.05
	CO_2_	0.00000635	0.0265	3.1765	6,740,000	256.34
WS_2_	Air	0.00000207	0.0631	1.035	2,200,000	225.76
	CO_2_	0.000153	1.51	76.50	162,000,000	9509.10
	O_2_	0.00000183	0.0568	0.915	1,940,000	201.34

## Data Availability

Not applicable.
